# Social Media Web 2.0 Tools Adoption in Language and Literacy Development in Early Years: A Scoping Review

**DOI:** 10.3390/children9121901

**Published:** 2022-12-03

**Authors:** Yiran Zhao, Jinjin Lu, Stuart Woodcock, Yuejing Ren

**Affiliations:** 1 Academy of Future Education, Xi’an Jiaotong-Liverpool University, Suzhou 215000, China; 2 School of Education and Professional Studies, Griffith University, Brisbane 4122, Australia; 3 School of Education Science, South China Normal University, Guangzhou 510631, China

**Keywords:** social media tools, language and literacy, child development in early childhood education, scoping review, pedagogical framework

## Abstract

Social media tools are increasingly used in child’s language and literacy development in early years. However, few researchers shed light on effectiveness and the practice that the EC professionals and teachers have adopted in ECE settings and other related contexts. This scoping review synthesized and evaluated the literature on social media integration in language and literacy development in ECE in the last decade, to provide a clearer picture on what social media tools were used, how they were used, and whether they were effective. Results showed that a wide-range of social media tools were used in diverse learning activities; however, few studies designed the learning activities with the guidance of an evidence-based teaching method or pedagogical framework.

## 1. Introduction

Web 2.0 refers to a perceived second generation of the Internet where users can create content, share, interact, and collaborate; it emphasizes a participatory culture among Internet users [1,2]. Compared with Web 1.0 where Internet users can only passively view content, Web 2.0 brings benefits of efficient multi-way communication among Internet users [2,3]. Supported by Web 2.0, social media are “interactive technologies that facilitate the creation and sharing of information, ideas, interests, and other forms of expression through virtual communities and networks” [4].

In recent years, social media Web 2.0 tools are increasingly used in language and literacy instruction (e.g., [1,5]). while also gaining momentum in early childhood education (ECE) [6]. ECE here refers to the care and education of children from birth to eight [7]. Although scholars have explored the use of social media in literacy practices and instructions in ECE [8,9,10], there is limited empirical evidence regarding the effectiveness of using social media on children’s language and literacy development. This scoping review will analyze and synthesize studies on using social media tools to support language and literacy development in early childhood education, and to demystify the characteristics of social media tools that are currently used in language and literacy development for young children; clarify the ways they are used; and, most importantly, synthesize evidence on their effectiveness.

### 1.1. Digital Technology in ECE

Although ECE has not been quick in taking advantage of the ubiquitous presence of [11] digital technologies [8], a variety of digital technologies have been used in ECE [12,13]. Some of the major technologies that are gaining momentum in ECE include e-books (e.g., [14,15,16], games (e.g. [17,18]), AI technologies (e.g. [18,19]), and mobile touchscreen technologies (e.g. [20,21]). Many of these technologies can be social media Web 2.0 tools or have social media features. For instance, e-book platforms that allow online sharing, collaboration, and interaction among the readers are social media Web 2.0 tools [22,23]. Online games in which players can communicate, collaborate, and interact with each other are also social media tools, such as Minecraft (education edition) [3]. Touchscreen mobile devices or tablets are often used in combination with applications. Lynch and Redpath [24] classified apps by their openness and closedness. Whereas closed apps follow a behaviorist approach to skill instruction by rewarding correct answers, open apps allow children to be creators and direct their own activities; open apps also allow connections to other apps or sharing of children’s creations [24]. All social media apps are open apps, but not all open apps are social media apps because allowing connections with other apps or sharing does not necessarily mean users can engage in online interactions in the app. It can be seen that a lot of knowledge about social media Web 2.0 tools adoption in ECE is masked under studies of tablets, games, e-books, or digital technologies in general.

However, social interaction is an important function and benefit that media Web 2.0 tools bring to early years education. Without social interaction and resource sharing, learning will not have any meaningful relationships among children [12]. In addition, children instinctively need to interact with others in meaningful ways and share what they have learned [25]. Therefore, social media is well-positioned to satisfy children’s needs to interact with others and share their learning with people potentially across the globe. Thus, there is an urgent need to comb through existing studies and synthesize available evidence on the use of social media Web 2.0 tools in language and literacy development in ECE, to shed light on the potential unique ways of utilizing social media for early childhood education.

### 1.2. Social Media Web 2.0 Tools in ECE

In early years, social media Web 2.0 tools have been most frequently used for parent–school communication; scholars found social media to be an efficient, flexible, and effective way to increase parental involvement, enhance mutual understanding and build partnerships between families and schools [8,26,27,28,29]. Meanwhile, social media Web 2.0 tools have also been used sporadically to assist young children’s learning and development in a variety of domains, such as media education [30], math learning [31], music education [32], health promotion [33,34], connected learning [35], higher order thinking skills [36], physical activities [6], and social competence [37]. However, these studies tend to focus on describing the practices and perceived benefits and challenges, with limited rigorous evidence given on actual gains in child learning and development. In addition, language and literacy development is particularly suitable for adopting social media Web 2.0 tools. This is because social media Web 2.0 tools enable children to communicate with real audiences in authentic tasks, so children can have a meaningful purpose for their literacy practices. The back-and-forth communications with audiences on social media platforms also give children motivations and directions for improving their language use and deepen their understanding of the functions of language [38]. Therefore, it is important to have a special focus on how researchers and educators have used social media Web 2.0 tools to promote language and literacy development in ECE.

### 1.3. Social Media in Language and Literacy Development in ECE

Many existing studies explored how social media Web 2.0 tools are used in language learning among older populations including upper primary school, secondary schools, and particularly college and adult learners [1,5]. Less studies have been conducted in early childhood. Before Web 2.0 was coined by O’Reilly in 2004, text-based technologies were the dominant technology used in language learning [1]. However, when using web 2.0-based social media tools in language learning, there is greater diversity in the type of tools involved. In Wang and Va ’squez’s [1] review, social text publishing tools including blogs and wikis were used in 58% of the studies.

When investigating social media integration in language and literacy development in ECE, scholars have primarily focused on describing how teachers used social media tools to structure their literacy instruction or play-based classrooms [8,10,39,40,41,42]. While some scholars zoomed in on one classroom to provide a rich description [8,39,41], others have intentionally explored multiple cases to illustrate the complexity around the digital transformation of early literacy instruction [10,40,42]. While most studies explored the learning of a dominant language, Peterson [41] explored using Skype to increase preschoolers’ opportunities to participate in minority language activities beyond school borders in a multilingual context.

In the home context, scholars have explored how social media was used to maintain heritage language [43,44,45] or to facilitate digital play to enrich literacy learning opportunities [46,47]. Scholars were also interested in deconstructing young children’s digital text creation process; using case studies, they examined how young children utilized digital resources for text production and the type of semiotic resources and strategies employed by the children [9,47].

After the outbreak of COVID-19, scholars have also paid particular attention to how social media Web 2.0 tools can be used to provide virtual learning opportunities [48,49]. These studies focused on describing teachers’ practices during virtual learning sessions, and often did not distinguish between practices that were used for language and literacy development and practices for child learning and development in other domains.

Overall, the existing research provided very rich descriptions and in-depth understandings of how social media tools were utilized to facilitate language and literacy development both in school and in the home in formal and non-formal learning. However, these studies tend to be case studies or used small samples; there is a lack of a more comprehensive picture on how social media tools are used to facilitate language and literacy development in young children, particularly given the complexity of digital transformation in different pedagogical contexts found in the literature [10]. More importantly, previous research had not focused on analyzing the effects of such practices on children’s language and literacy skills. Limited research is known about whether the diverse social media-mediated language and literacy learning practices could really lead to gains in children’s language and literacy skills.

This scoping review provides critical information for early childhood educators and researchers on the use of social media in language and literacy development, and its effectiveness. This review will shed light on enhancing practice and evaluation of social media tool integration in early childhood language and literacy development.

### 1.4. Research Objectives and Questions

This review aims to evaluate, synthesize, and display the latest literature on using social media tools to support language and literacy development in ECE settings. It is intended to analyze and present information on the research design, social media tools used, learning activities involved, and effects of social media integration on language and literacy development of young children in ECE settings. To achieve these objectives, we focused on studies that examined the application of social media tools to support language and literacy development in ECE. In addition, this study puts forward possible pathways for future research on social media tools in ECE, hoping to establish a strong theoretical basis and clarify challenges that might hinder the effective application of social media tools in ECE.

This review is guided by the following research questions (RQs):

RQ1: What social media tools were used to support language and literacy development in ECE?

RQ2: How were social media tools used to support language and literacy development in ECE?

RQ3: What were the effects of using social media tools on language and literacy development in ECE?

RQ4: What were the research methods used in studies that examined the implementation of social media tools in language and literacy development in ECE?

## 2. Methods

The methodology of this scoping review follows the framework established by Arksey and O’Malley [50], and Levac et al. [51]. This scoping review went through a five-stage process involving identifying research questions, identifying relevant studies, study selection, data charting, and reporting results.

### 2.1. Information Sources and Search Strategies

The electronic databases used for the literature search included Education Resources Information Center (ERIC), Web of Science, EBSCO, Psych-Info, Medline, and PubMed. Search terms were discussed among the research team and used in combination: (child OR infant * OR “early child *” OR “early years” OR toddler OR preschool *) AND (educator OR teacher OR mentor) AND (play * OR interact * OR converse * OR language OR talk OR communicate * OR cooperate *) AND (digital technology OR social media * OR Web 2.0 OR digital tools OR high technology *). The search was limited to peer-reviewed journal articles in English that were published from January 2012 to mid-2022 when the search was conducted. All articles were accessed from July to August 2022.

### 2.2. Inclusion and Exclusion Criteria

Table 1 below outlines the inclusion and exclusion criteria applied in study selection.

### 2.3. Study Selection

**Figure 1:**
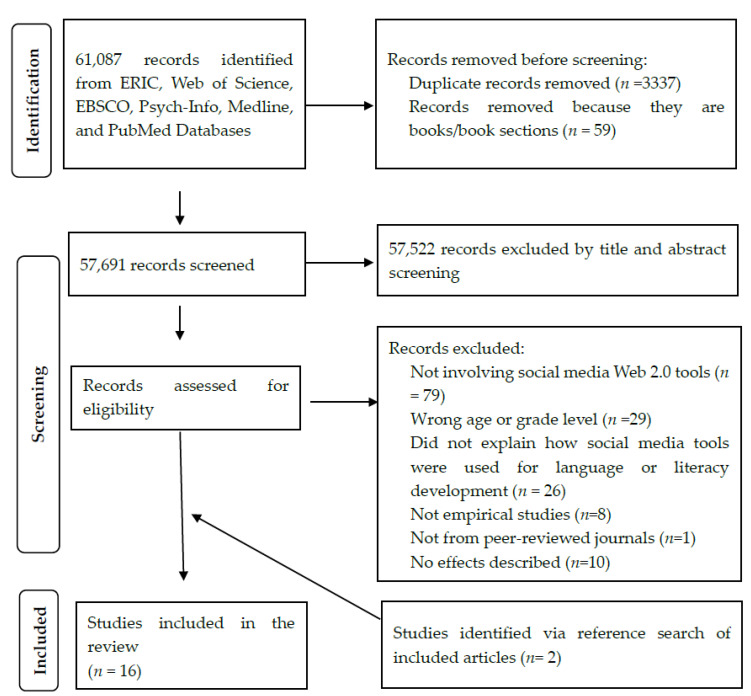
PRISMA diagram of included articles in the scoping review.

As shown in Figure 1, a total of 61,087 records were found using our search strategy. A total of 1348 articles were found on Web of Science, 357 on PubMed, 288 on Medline (searched via EBSCO), 767 on Psych-Info (searched via EBSCO), 54,253 on ERIC, 4074 on EBSCO. Overall, 3337 duplicates were identified and removed using Endnote 20’s duplicate detection function. Articles were removed because: (1) they were not relevant to the research topic based on title and abstract (*n* = 57523); (2) they did not involve social media Web 2.0 tools (*n* = 79); (3) children examined in the study were of wrong age or grade level (*n* = 29); (4) they did not explicitly explain how social media tools were used for language and literacy development (*n* = 25); (5) they were not empirical studies (*n* = 8); (6) they were not from peer-reviewed journals (*n* = 1); (7) they did not provide evidence on the impact of using social media on language and literacy development (*n* = 10). We also searched the references of the included studies and some relevant literature review studies and identified another two articles for inclusion. Inconsistencies between the co-authors in the study selection process were resolved through discussion. In total, 16 studies were thoroughly examined in this review.

### 2.4. Analysis

Title, keywords, and the main text of the included studies were used for the analysis. The co-authors generated the coding scheme and an excel-based data extraction chart in discussion. Then, data extraction was carried out by the primary researcher independently while the second author audited the data charting results.

## 3. Results

Despite its importance, the topic of social media tools integration in language and literacy development in ECE is under-studied; only 16 studies could be identified in this review. Among the 16 studies, 8 were conducted in the USA. The rest were conducted mostly in developed countries including Sweden (2 studies), Greece, UK, and Canada (1 study). Furthermore, in Brazil, Turkey and China, there was one study completed in each county. Twelve studies were conducted in the school setting [25,38,52,53,54,55,56,57,58,59,60,61] while four studies examined the home setting [62,63,64,65]. In particular, three studies actually emphasized home–school collaboration. Dore at al. [52] examined a virtual summer kindergarten readiness program during the COVID-19 pandemic. Meanwhile, both Snell et al. [64] and Olszewski and Cullen-Conway [65] used social media tools to provide parents with instructions on home literacy activities, and then proceeded to evaluate the effectiveness of such practices on children’s emergent literacy development. More basic characteristics of the included studies including research aims, research design, participants, instruments, and target language are available in Table 2.

### 3.1. What Social Media Tools Were Used to Support Language and Literacy Development in ECE?

There is great diversity in the social media tools employed by the included studies. The 16 included studies employed 17 different social media tools. The only Web 2.0 tools that were used by more than one study were Google Docs and Twitter. Because of the diversity of the individual social media tools included, it might be beneficial to analyze what type of social media tools they represented. [3] provided a classification of social media tools; it outlined 14 social media types defined by the common characteristics of the social media tools that fell under each type.

Text publishing tools refers to tools primarily used for publishing texts such as stories, essays, conversations, and even presentation slides [3]. Text publishing tools were most frequently utilized by the included studies (*n* = 7). For instance, Weblog Typepad was a class blog created by the teacher for students to post writings and exchange comments [38]. Another example was ePearl which was a web-based digital portfolio tool which mainly provided the students with a text editor and an audio recorder to write down and share their ideas; however, students could also upload other file formats including images, photos, slideshows, etc. [58]. Interpersonal social media tools are tools for interpersonal communication online [3]. It was the second most frequently used type of social media (*n* = 5). For instance, online video chat services including Skype, Zoom, and Facetime had all been utilized in included studies. Productivity tools, which were defined as tools to enhance the organization’s productivity, were the third most often used type (*n* = 2). For example, Google Docs is a cloud-based word processor that allows online collaboration [55,56], while Google Slides is a cloud-based slide editor that allows collaboration and online presentation [60]. Audio publishing tools are tools primarily used for publishing and sharing audio segments [3]. For example, Papa is an audio publishing app where users can create and share audio files or music of up to 6 min long; the app also allows other users to leave an audio comment [61]. Audio publishing tools appeared in two studies while microblogging tools (Twitter) also appeared in two studies. Social gaming tools (Minecraft) were only used in one study. Table 3 provides a full list of the social media tools included, their key features, the social media type that each tool represents, and web links to official sites, if available.

Among all the social media tools included, five could also count as mobile social media tools as they either operate entirely as a mobile app (e.g., Book Creator app) or their mobile app version was used (e.g., Showme app). Other social media types were not found in the included studies, including social communities, photo publishing tools, video publishing tools, live casting tools, virtual world tools, really simple syndication, and aggregators. Please note that one social media tool could fall into multiple classifications, as social media tools today continuously expand their functionalities; not to mention that Van Looy’s [3] classification was not mutually exclusive.

While almost all studies used different social media tools, three studies used more than one social media tool. Snell et al [64] intentionally gave teachers freedom in choosing the app they preferred. Genlott and Gr$\ddot{o}$nlund [56] used different social media tools to achieve different purposes; whereas Google Docs was used for collaborative writing and providing formative feedback on work-in-progress, Google Sites was used to publish the final product to a wider audience for final comments. In Miller [25], both social media apps were used to share students’ work, but Showme app allowed students to create and share voice-over presentations with the same app on their own, while the teacher shared links to student work regularly on the class Twitter account.

### 3.2. How Were Social Media Tools Used to Support Language and Literacy Development in ECE?

We explore this research question from four perspectives: first or second language, dominant or minority language, specific language and literacy skill targeted, and learning activities involved.

Most studies used social media tools to support the learning of the dominant language in the country where the study was conducted. Among them, the dominant language was the first language of the children in eight studies [53,55,56,58,59,60,62,65]. Two studies examined a mixture of first-language learners and second language learners [52,64], while two studies focused on second language learners who were trying to acquire the dominant language [25,38].

Social media tools were used to support the learning of the minority language, in the context where the study took place, in two studies. While Eubanks et al [54] examined a Chinese immersion program in the school setting, Szecsi & Szilagyi [63] examined how social media was used to maintain heritage language at home. In two other studies, social media tools were used to support the acquisition of English as a foreign language in Turkey and China [57,61].

Writing was the most often targeted skill (nine studies) when integrating social media tools into literacy instruction, followed by reading (six studies), vocabulary (six studies), speaking (three studies), story comprehension (two studies), listening (two studies), narrative skill (two studies), letter knowledge (one study), phonological awareness (one study), reflective skill (one study), and print awareness (one study). One study did not report on a particular literacy skill as its target learning domain [25].

Social media tools were integrated into diverse learning activities. In the school context, the most common learning activity type involves the creation of digital texts or digital artefacts and sharing them online. Students might learn to write different genres by writing and publishing on social media platforms [38,55,56]; they might also be invited to document and reflect on their learning, including class activities, project findings, or homework assignments either via a single digital artefact or via an e-portfolio [25,58,59,60,61]. They might also create and publish a multimodal digital story [54]. Although sharing via online publishing is a unique feature of social media, social media can also allow online collaborations. However, only two studies intentionally utilized the online collaboration features of social media tools [55,56].

The Ellison and Drew [53] study was unique in that Minecraft was used to help students visualize what they would write about. During the COVID-19 pandemic, social media tools were also used to conduct online synchronized lessons both between the teacher and the child, and between the teacher and the parent [52]. In two studies, students not only used social media platforms for teacher-initiated learning activities, but also voluntarily created digital artefacts and texts and posted on the social media platforms [58,59].

Among the studies in the school context, only four studies reported a clear framework behind their pedagogical design. Genlott and Gr$\ddot{o}$nlund [55,56] used sociocultural theory to guide their pedagogical design of the Write to Learn method which used social media to enable collaborative writing, formative assessment, and formative feedback among peers. The 2013 study was a pilot of the 2016 study and the pedagogical design was refined in the 2016 study. The study by Eubanks et al. [54] used a pedagogy of multiliteracies and incorporated the three communication modes (interpretive, interpersonal, presentational) specified in the Standards for Foreign Language Learnings in the US as a design framework for its 21st century Chinese language writing workshop. Finally, the social media platform used in the study by Lysenko and Abrami [58] was designed under the theoretical framework of self-regulated learning. When using the platform, students would follow the steps of self-regulated learning including setting goals, completing the activity, and writing reflections.

When used in the home context, social media tools were often used for virtual story-time [52,62,63] or for providing parents instructions on literacy practices at home [64,65]. Dialogic reading was the only evidence-supported method that some of these programs reported to have employed [62,65]. For a full list of learning activities that were implemented using social media tools in the included studies, please refer to Table 4.

### 3.3. What Were the Effects of Using Social Media Tools on Language and Literacy Development in ECE?

As shown in Table 5, although all of the included studies explicitly reported on the impact of using social media tools in language and literacy development, only 11 studies measured the effects of integrating social media tools on language and literacy skills of young children, and the results were largely mixed [52,53,54,55,56,58,59,61,62,64,65]. Literacy instructions incorporating social media tools were found to have positive effects on young children’s alphabet knowledge, vocabulary, speaking fluency, story comprehension, reading skills including reading comprehension, and writing skills [52,54,55,56,58,61,64,65]. Several case studies or qualitative studies also provided some evidence on the positive impact of literacy instruction using social media tools, on children’s language and literacy development. However, these were mostly accounts based on teachers’ and parents’ perceptions or based on teachers’ or the researchers’ subjective evaluation of students’ written work, without providing very clear information on the evaluation standards and procedures used [25,38,57,60,63]. Among all the types of social media tools that were used in the included studies, productivity tools were consistently associated with improvements in children’s language and literacy skills [55,56,60]. Additionally, the productivity tools used all belonged to Google Apps for Education. Text publishing tools were also consistently associated with enhancements in children’s language and literacy skills across studies [38,54,57,58]. Although Lysenko and Abrami [58] only found positive effects on vocabulary, reading comprehension, and written expression, they did not find this on listening comprehension. The only exception was the Silvia de Oliveira et al. [59] study where evidence of the impact on language and literacy skills was inconclusive. Similarly, microblogging tools were reported to have a positive impact in both studies that utilized this type of tool [25,65]. However, in the Olszewski and Cullen-Conway [65] study, the positive impact was only found on vocabulary and story comprehension, but not on print knowledge. The findings related to interpersonal social media tools were more mixed. While most studies reported positive effects on a selected range of language and literacy skills among all the skills they assessed [52,63,64], Gaudreau et al. [62] actually found no differences in students’ reading comprehension among the groups that used social media tools and the groups that did not use such tools. Audio publishing tools had a positive impact on children’s speaking fluency but were only utilized in one study [61]. So, productivity tools, text publishing tools, and microblogging tools seem to be the types of social media tools that were more consistently associated with improvements in young children’s language and literacy skills.

### 3.4. What Were the Research Methods Used in Studies That Examined the Implementation of Social Media Tools in Language and Literacy Development in ECE?

This section outlines a summary of the research methods used in the studies that examined the integration of social media tools in language and literacy development in ECE.

Quasi-experimental design was most frequently employed (six studies) but studies varied with regards to the inclusion of control groups and pretests. The pretest posttest with non-equivalent groups design was used by three studies [55,58,61]. Two studies did not include a control group [52,65] while Genlott and Grönlund [56] switched to a design with a control group but no pretest because this time they used the National Standard Literacy Test in Sweden to measure literacy skills; the test could only be taken by 3rd graders while their intervention started in 1st grade.

Case study and multi-case study was the second most frequently (four studies) used research design [38,53,59,60]. Triangulation of data sources can be found in most of the case studies. For instance, Silvia de Oliveira et al. [59] used observation, child assessment, and analysis of student-created digital artefacts to examine the changes in students’ reading and writing practices after receiving a laptop with internet access. Theodosiadou and Konstantinidis [60] also used a mixed-methods data collection method including a parent questionnaire, teacher interviews, and analysis of students’ writing samples to examine the impact of e-portfolio on learning in a Greek primary school. Other case studies only collected qualitative data, but still used more than one source of data; the most commonly used sources of data include observations, interviews, and students’ work samples [38,53].

Three studies employed a qualitative research design; data collection was conducted by interview [57] or teachers’ narrative accounts [25], or a combination of interview and auto-ethnographic interview [63]. Two studies used randomized experiments [62,64] and one additional study used a mixed-methods design and collected data via observation, surveys, and audio-visual materials [54].

## 4. Discussion

Social media has been increasingly used in ECE, particularly in children’s language and literacy development. Scholars have extensively described how social media tools are used in literacy instruction in the school and literacy practices at home. However, little evidence-based research showed the extent to which young children’s language and literacy skills were gained when social media tools were adopted. To address this gap, this article provides a scoping review of studies on integrating social media tools into language and literacy development in early childhood, focusing on the features of social media tools used, implementation of social media tools in literacy learning activities, program effectiveness, and study design. Despite the limited number of empirical studies that explicitly provided evidence on the impact of integrating social media in language and literacy development in early childhood, new insights were gained from the existing references into what and how social media tools were used in the language and literacy development of young children, and their effectiveness. Moreover, addressing the important issue of integrating social media tools in language and literacy development in ECE generates new opportunities for understanding how to best utilize social media tools to support young children’s language and literacy development, and the benefits it might bring to children’s linguistic development.

This scoping review showed that very few studies provided evidence on the impact of social media use on language and literacy development in early childhood, and on children’s language and literacy outcomes. Merely 16 studies could be identified. Most of the studies were conducted in developed countries except for the three studies that took place in Turkey, Brazil, and China. Future studies could focus more on developing countries, as young children from less developed regions may also benefit from the use of social media in early childhood education.

This review has showcased a variety of social media tools that were utilized for language and literacy development in early childhood. A total of 17 different tools were used in 16 studies with only Google Docs and Twitter appearing in more than 1 study. Although previous studies have provided some guidelines on choosing educational apps for preschoolers, especially apps designed for literacy development [66,67,68], the apps they evaluated were not all social media tools, and not all social media tools are apps. Therefore, the framework for evaluation of apps provided by these studies may not necessarily address the unique strengths of social media tools in ECE, or cover the non-app-based social media tools. Future studies should evaluate and compare the strengths and weaknesses of these social media tools and develop a framework for choosing social media Web 2.0 tools for language and literacy in ECE, to ease the selection by practitioners, caregivers, and researchers.

Among the various social media types identified by Van Looy [3], five out of the seventeen tools examined were mobile social media tools. This is consistent with the increasing trend of using touchscreen mobile devices in ECE [20]. Text-publishing tools, interpersonal social media tools, and productivity tools were the top three most prevalently found social media tool types in the included studies. Audio publishing tools, microblogging tools, and social gaming tools were also present but with a lower frequency. The included studies did not experiment with social communities, photo publishing tools, video publishing tools, live casting tools, virtual world tools, Really Simple Syndication, and aggregators. However, these types of social media tools also boast great potential for language and literacy development. For instance, previous studies have found positive effects in using Facebook, a social community tool, to enhance language and literacy skills in adult learners and even students with dyslexia [69,70]. Both live casting tools and virtual world tools can facilitate remote literacy learning as the COVID-19 pandemic continues, and the virtual world tools such as Second Life can create a more immersive experience than regular interpersonal social media tools such as Skype. YouTube, a video publishing tool with Really Simple Syndication functions, has also been used to facilitate heritage language maintenance and digital play out of school [44,46]. Future studies should examine the effects of using these less-studied social media tool types, on language and literacy development in early childhood.

Among the various types of social media tools utilized in the included studies, productivity tools, text publishing tools, and microblogging tools are the types of social media tools that were more consistently associated with improvements in young children’s language and literacy knowledge and skills. However, this finding should be interpreted with caution for two reasons. First, some types of social media tools such as the audio publishing tools were only used in one study; therefore, there were simply not sufficient amounts of evidence to draw conclusions on whether audio publishing tools can be consistently associated with language and literacy skill improvements. Second, the number and type of language and literacy knowledge and skills assessed varied across studies. When studies assessed more diverse knowledge and skills, they were more likely to find positive effects to be restricted to a selected range of knowledge and skills, compared to studies that only assessed one type of language and literacy skill. Therefore, future studies should further examine the less-studied social media tools, echoing the point above. Future studies should also measure a wider range of language and literacy knowledge and skills.

Social media tools were used for both first and second language acquisitions and both dominant and minority language acquisitions. Only two studies examined foreign language acquisition [57,61]. Yet, previous studies have found social media to be effective for foreign language learning among upper-primary, secondary, and college level learners, as it enables learners to go beyond borders [1,5]. Future studies should explore more on the use of social media tools for foreign language learning in the context of ECE.

Writing and reading were the most often targeted language skills when using social media for language and literacy learning for young children. This review revealed that learning activities that involved social media tools were diverse. Social media was most frequently used for creating and publishing digital texts and artefacts. By publishing children’s digital creations online to a wider audience, teachers were able to provide the students with an authentic and meaningful purpose for their creations [38]. This was a main purpose for social media integration in literacy instruction among the included studies and a reason why reading and writing skills were the most often targeted skills. However, this does not mean that other emergent literacy skills could not be improved with the help of social media. More studies on how social media tools may be used to develop other emergent literacy skills, is warranted in future studies. Moreover, the wider audience was carefully selected to be composed of only other students, teachers, and families, thereby creating a safe online interaction environment for the children while fostering better family–home connections [25,38]. Future studies might want to compare the effects of different audience composition on language and literacy development when using social media tools.

Among all the included studies, only six studies had a theoretical framework for pedagogical design that informed their learning activities. When integrating digital technologies in ECE, paying too much attention to the tools over the pedagogy can be a problem [7]. Future studies should pay closer attention to pedagogical design.

Most studies employed a quasi-experimental design, followed by case study design, qualitative design, randomized experiment, and mixed-methods design. The study samples were also relatively small in most studies. Thus, it is unclear whether the findings were generalizable to other populations and contexts. Future studies should consider following Genlott and Grönlund’s [55,56] example to first rigorously test the effectiveness of a social media integrated pedagogy in a smaller sample, and then validate the results with larger samples.

Moreover, out of the 16 studies included, only 11 studies measured the language and literacy skills of the children after using social media to support language and literacy development, and the results were mixed. Several studies did not use instruments that were rigorously designed and validated [53,54,55]; in some studies, the data were not rigorously analyzed [54,59]. The other five studies only used parental perceptions, teacher perceptions, or teachers’ and researchers’ subjective evaluations as evidence for the positive impact on literacy development, while providing very limited information on the evaluation procedures and standards followed [25,57,60,63]. This will necessarily make any positive findings less convincing. Future work should provide more empirical evaluations of the effects of social media integration in language and literacy development in early childhood and consider using psychometrically sound measurements to assess child outcomes, and to better guide future practice.

### 4.1. Limitations and Contributions

This scoping review can inform future research in advancing social media tool selection, research design, assessment of language and literacy skills, learning activity design when incorporating social media tools in the language, and literacy instruction in early childhood. It can also provide researchers and practitioners with some guidance on the design, implementation, and evaluation of age-appropriate social media tools for young children. However, this scoping review is exploratory in nature, given the small number of eligible studies on the research topic of social media integration in early childhood language and literacy development. Therefore, more advanced techniques such as meta-analysis and systematic review were not used. Several included studies were focused on tablet use, digital play, or general information and computer technology (ICT) integration. They did not provide detailed descriptions of the functionalities of the apps or technological tools incorporated in their study. The authors had to manually check the official websites of these tools and determine whether such tools are social media tools based on the definitions provided in this study. In addition, this study only explored journal articles published in peer-reviewed journals, and did not incorporate grey literature, books, and organizational reports. Thus, some social media tools, or studies that employed social media tools, may have been excluded from this study. Finally, this study only included studies published in English; thus, studies published in other languages may have been neglected. Future researchers should organize multi-cultural teams to examine publications in more diverse languages. Nevertheless, this scoping review could provide valuable directions for integrating social media and Web 2.0 tools in early childhood education and serve as a reference for future research on technology integration in ECE.

### 4.2. Future Research Direction

Most of the included studies were conducted in Western contexts except Sun et al. [61]. Moreover, first-language learning was more often examined than second-language acquisition and more studies used social media tools to support dominant language acquisition rather than minority language learning. Using a culturally responsive approach to language learning in early childhood warrants the examination of more contexts, more second language learners, and more minority language learning in future studies.

In several studies, multiple social media tools were used together [56] or social media tools were used in combination with other technologies such as mobile applications that are not social media, e-book platforms without social media features, and interactive whiteboards [54,58,59]. Future studies should compare and contrast the pedagogical affordances provided by different combinations of social media tools with additional social media tools, or other non-social media technologies commonly found in early childhood classrooms.

Among the studies that used social media in school settings, four studies used social media tools to create homework assignments or project assignments that extended the learning beyond the classroom [57,58,59,61]. Future studies could compare the effectiveness of integrating social media tools in class activities, versus using it to extend learning beyond the classroom.

Young children in the included studies also exhibited improvements in reflections on their learning. While some researchers chose tools or pedagogical designs that could intentionally foster self-regulated learning abilities in young children [58,60], other researchers also witnessed children’s voluntary reflections on their learning [59]. Future studies could analyze how self-regulated learning is fostered as a byproduct of social media integration in literacy instruction, and whether the enhanced self-regulated learning ability of young children further contributes to gains in language and literacy skills.

In this review, only four studies examined using social media tools for language and literacy development in the home context, and three of them provided rigorous evidence for impact on language and literacy skills [62,64,65]. However, there are additional previous studies that examined social media integration in language and literacy practices at home, without assessing its impact on child learning [44,45,46,47]. Future studies could provide more evidence on the impact of using social media at home on language and literacy development of young children.

### 4.3. Implications for Policy and Practice

Although a limited number of studies measured the impact of social media integration on young children’s language and literacy skills, they did find social media tool integration in language and literacy instruction to have positive effects on young children’s alphabet knowledge, vocabulary, speaking fluency, story comprehension, reading skills (including reading comprehension), and writing skill [52,54,55,56,58,61,64,65]. Moreover, they also reported improved students’ attitudes towards writing [54], learning engagement and confidence in creative writing [53], and motivation and commitment [60]. Therefore, social media integration in language and literacy instruction in early years should be promoted at both fronts of policy and practice.

When teachers devise their own ways to integrate social media into language and literacy instructions, teachers should also devise indicators of child progress to guide the refinement of the practice. Policymakers and researchers could invent rigorous and easy-to-use evaluation tools that can help teachers better gauge the effectiveness of their self-invented practices. Although evidence on effectiveness of these practices is not consistently rigorous across studies currently, there are some pedagogies that have been rigorously developed and validated such as the Write to Learn method [55,56]. Teachers could also adapt such practices for their own teaching instead of devising something new.

There are a variety of social media tools but not all of them are available in multiple regions, or multiple languages, or on all types of devices. This review is not meant to recommend a specific social media tool to teachers; rather, it aims to help teachers understand the types of social media tools available and what learning activities they enable children to engage in. When choosing social media tools for instruction, teachers should first clarify their purposes for social media tool integration and then determine which specific social media tool best serves their instructional purposes. It might be necessary to combine multiple social media tools or combine social media tools with other technologies, just like several studies did to achieve the instructional purposes [54,58,59].

Moreover, social media integration can be effectively implemented without achieving one tablet/laptop per child [38,56]. This knowledge has particularly important implications for early childhood educators in less developed countries/regions and socioeconomically disadvantaged populations.

Finally, several studies found that the use of social media tools in literacy instruction effectively improved parental involvement and parent–school communication [25,38,64]. So, using social media in literacy instruction in early years can promote mutual understanding between the school and the parents, and contribute to a school–family partnership model in early childhood education and care.

## 5. Conclusions

Social media tools are increasingly incorporated into language and literacy development in ECE settings. However, limited evidence is available on whether such practices are effective at achieving their goals: enhancing young children’s language, and literacy development. This review synthesized and evaluated the most updated literature on social media integration in language and literacy development in ECE to provide a clearer picture on what social media tools were used, how they were used, and whether they were effective. Results showed that a wide-range of social media tools were used in diverse learning activities; however, few studies designed the learning activities with the guidance of an evidence-based teaching method or pedagogical framework. Moreover, existing studies presented mixed findings on whether social media integration was effective in enhancing young children’s language and literacy development. Only eight out of the sixteen studies included provided evidence of positive effect. More importantly, most studies were fraught with methodological difficulties, including the lack of rigorously designed and validated instruments, evaluation procedures, evaluation standards, and rigorous data analysis methods, thus making the positive findings less convincing. To advance the research and practice on social media tool integration in language and literacy development in ECE settings, more rigorous evaluations of the effectiveness of social media tool integration are necessary, and the results of such evaluations should be used to guide the design of evidence-based learning activities both at home and in school.

## Figures and Tables

**Table 1:** Criteria for inclusion and exclusion.

Criterion	Inclusion	Exclusion
Age of participants or children taught by the participating teachers or children in the participating families	Age range of 3–8 years old, or children attending preschool to 3rd year in primary school if age is not reported	Children in other age range or grade range
Mental and physical fitness	Children who are mentally and physically fit	Children who are not mentally or physically fit
Literature focus	Using social media tools to support language and literacy development, with evidence on the impact of such practices on children’s language and literacy development	Studies not involving social media tools and studies that did not explicitly explain how social media tools were used for language and literacy development, and studies without evidence on the impact of such practices on children’s language and literacy development
Article type	Full-text, empirical research from peer-reviewed journals	Articles that are not empirical research and articles that are not from peer-reviewed journals
Language	English	Non-English
Publication Date	1 January 2012 to 31 August 2022	Before 1 January 2012

**Table 2:** Characteristics of included studies.

Author (Year)	Research Aims	Design	Instruments	Participants	Country	Target Language	Setting
Dore et al. (2021) [52]	To assess the feasibility, social validity, and preliminary impacts of a virtual summer kindergarten readiness program during the pandemic	Quasi-experimental: pretest and posttest design, no control group	Child assessment	A total of 91 caregivers and their preschool-aged children with an average age of 63 months (SD = 4, min = 53, max = 72). Overall, 73% of the participants spoke English at home, while 27% of the participants were English language learners	USA	English	School
Ellison & Drew (2020) [53]	To examine whether and how digital play via sandbox games can support creativity in boys’ writing	Case study	Focus group interview and evaluation of student writing samples	A total of 12 Year 3 boys in one classroom undertook the intervention; 8 participated in interviews and 6 agreed to have their written work be analyzed	UK	English	School
Eubanks et al. (2018) [54]	To explore the effect of a technology integrated 21st century writing workshop on the ability and attitude towards writing of children enrolled in a Chinese language immerision program	Mixed-methods	Observation, pre-survey, post-survey, audio-visual materials collection	24 second-grade students (7–8 year-olds, 7 boys, 17 girls) enrolled in a Mandarin Chinese program in Colorado. Most participants are non-native Chinese speakers; only one child was a Chinese heritage speaker because her parents spoke Chinese at home	USA	Chinese	School
Gaudreau et al. (2020) [62]	To compare the effects of dialogic reading over video chat and more traditional forms of book reading, in promoting story comprehension and vocabulary learning	Randomized experiments with three experimental conditions: (video chat vs. live vs. prerecorded book reading)	Child assessment	A total of 58 4-year-olds in the three experimental conditions and 11 children in the control condition where they only completed the pre-tests and post-tests without reading the book	USA	English	Home
Genlott & Gronlund (2013) [55]	To test the effectiveness of a new method of learning to read and write in early years	Quasi-experimental: pretest and posttest design with non-equivalent groups	Child assessment and student work samples	A total of four first-grade classes in the same school; the children were 7-year-olds. Two classes which contained 41 students were in the experimental group and two classes which contained 46 students in total were in the control group	Sweden	Swedish	School
Genlott & Gronlund (2016) [56]	To compare the effectiveness of an ICT-integrated pedagogical method, Write to Learn (WTL), with traditional pedagogy or using ICT without WTL, in literacy development and mathematics	Quasi-experimental: posttest only design with non-equivalent groups	National Literacy Test for grade 3	A total of 502 grade 3 students in a Swedish city who started to experience the WTL method from grade 1	Sweden	Swedish	School
Kaynar et al. (2020) [57]	To examine teacher perceptions on e-book use in their classroom	Qualitative	Interview	A total of 13 English teachers in 13 different classrooms containing children aged 4 to 6-years-old from three different campuses of a private chain preschool/kindergarten.	Turkey	English	School
Lysenko & Abrami (2014) [58]	To explore the impact of integrating two web-based applications into early elementary students’ literacy instruction on their reading comprehension	Quasi-experimental: non equivalanet groups, pretest and posttest design	Child assessment	In study 1 in 2010–2011, 351 students from 22 classes and their teachers were included in the analysis. Among them, 114 grade 1 and 61 grade 2 students were in the experimental group. In study 2 in 2011–2012, 166 students in 10 classes participated. Among them, 27 grade 1 and 67 grade 2 students were in the experimental group. The experimental group included four teachers who were all continuing on from study 1; another five control teachers were newly recruited.	Canada	English	School
Miller (2018) [25]	To examine the effect of emerging technologies on English language acquisition for native Spanish-speaking ELLs enrolled in ESL and bilingual education preschool and elementary programs	Qualitative	Teachers’ narrative accounts	A total of 46 bilingual and ESL teachers in early childhood centers or elementary schools who self-perceive as innovative technology users from five Texas public school districts in the Gulf Coast region, central Texas region, and Dallas-Fort Worth metropolitan area	USA	English	School
Szecsi & Szilagyi (2012) [63]	To explore immigrant Hugarian families’ perceptions about the role media technologies play in their child’s development, maintenance of heritage language, and cultural identity	Qualitative	Interview and autoethnographic interview	The study included two families. Only family one had a boy aged five who fell into the age range of this review. So only his data were charted for this review	USA	Hungarian	Home
Shin (2014) [38]	To examine how ELL students use blogging to develop academic literacy and how does this process shape the social, political, and academic nature of students’ literacy practices	Case study	Observation, interview, informal conversations, documents and materials (written texts, blog postings, instructional materials, and school documents).	One Spanish-speaking 2nd grade student who did not have internet-related computer experience prior to the research project. The whole project was conducted with 20 2nd graders in one 2nd grade class where the majority of the students’ primary language was Spanish, but this study only reported on one student	USA	English	School
Silvia de Oliveira et al. (2013) [59]	To investigate changes in students’ reading and writing practices after receiving full access to a laptop with internet connections	Case study	Observation, child assessment, student-created digital artifacts	A total of 19 6-year-old students in a 1st grade classroom and their teacher in a public school which participated in the One Laptop per Child project	Brazil	Portuguese	School
Snell et al. (2022) [64]	To investigate the impact of a vocalbulary-focused texting program on home-school connections in preschool children’s vocabulary and language learning.	Randomized experiments	Child assessment and survey	A total of 173 treatment children (24 lead teachers) and 173 control children (25 lead teachers), and their parents or guardians from public school prekindergarten and Head Start programs in a large East Coast city. Children aged 3–5 years old	USA	English	Home
Theodosiadou & Konstantinidis (2015) [60]	To examine e-portfolios’ impact on learning in a Greek primary school environment	Case study	Survey, interview, and student work samples	A total of 14 8-year-old pupils in 3rd grade in a public primary school in northen Greece	Greece	Greek	School
Sun et al. (2017) [61]	To examine the effectiveness of integrating mobile SNS with face-to-face instruction in improving speaking skills of young children	Quasi-experimental design: pretest and posttest with nonequivalent groups	Child assessment	A total of two 1st grade English classes (average age 6.5 years) in an urban public elementary school in Beijing. The children had prior experiences using iPads in learning English as iPad was integrated into the curriculum. One class was the experimental group (37 children, 17 girls and 20 boys), one class was the control group (35 students, 14 girls and 21 boys)	China	English	School
Olszewski & Cullen-Conway (2017) [65]	To investigate whether social media platforms can be used to promote parent–child literacy behaviors in families with pre-school children	Quasi-experimental design: one group pretest-posttest design	Child assessment and observation	A total of seven children of preschool age and their parents	USA	English	Home

**Table 3:** An overview of the social media tools used in the included studies.

Social Media Tool (Number of Studies That Used This Tool)	Key Features	Type	Web Link
Google Docs via Google Drive (*n* = 2)	A cloud-based word processor that allows collaboration.	Productivity tools	https://www.google.com/docs/about/ (accessed on 30 August 2022)
Twitter (*n* = 2)	A microblogging platform.	Microblogging tools	https://twitter.com/ (accessed on 27 August 2022)
Google Slides via Google Drive (*n* = 1)	A cloud-based slide editor that allows collaboration and online presentation.	Productivity tools	https://www.google.com/slides/about/ (accessed on 30 August 2022)
Google Sites (*n* = 1)	A platform for creating one’s own site without any programming skills.	Text publishing tools	https://workspace.google.com/products/sites/ (accessed on 30 August 2022)
Book Creator appp (*n* = 1)	A digital book-making tool. Children can use texts, images, videos, audios, color, and movements to create digital books. Students can embed contents from other apps such as Google Maps or Youtube videos. Each teacher can create a class library for children to publish their books. Created books can also be shared in various ways including hard print, email, ibooks, or shared with an assigned code to Google Drive. It also allows real-time collaboration among multiple users and it can read the student-created book aloud to the audience.	Text publishing tools Mobile social media tools	https://bookcreator.com/ (accessed on 27 August 2022)
Skype (*n* = 1)	A video chat service.	Interpersonal social media tools	https://www.skype.com/en/ (accessed on 30 August 2022)
Minecraft education version (*n* = 1)	A sandbox game with no set objectives; using a block-like structure, players can create freely at their own will, similarly to LEGO in the virtual world. The educational version allows teachers to control many in-game features such as chat features, fighting, weather and player’s health so that the students can concentrate on the educational element of the game.	Social Gaming tools	https://www.minecraft.net/ (accessed on 28 August 2022)
ePearl (*n* = 1)	A web-based digital portfolio tool that encourages students to set goals, device and monitor strategies to achieve their goals, and to reflect on their learning. Work can be shared with teachers, peers, and parents to obtain feedback. It offers both a text editor and an audio recorder, slideshows, videos, podcasts, scanned images, and photos can also be uploaded to the platform to document student work.	Text publishing tools	https://www.concordia.ca/research/learning-performance/tools/learning-toolkit/epearl.html (accessed on 27 August 2022)
Raz-Kids (*n* = 1)	An interactive e-book platform. Key features include interactive, levelled e-books and accompanied eQuizzes, assessment of student reading levels, student incentives and awards, student avatars, student management portal for teachers, student and teacher autonomy in choosing which materials to use. Its social media feature is that the teacher can comment on student work via writing or recording.	Text publishing tools	https://www.raz-kids.com/ (accessed on 29 August 2022)
Zoom (*n* = 1)	A video conferencing platform. Key features include meetings, cloud phones, webinars, access and use other apps from within Zoom.	Interpersonal social media tools	https://zoom.us/ (accessed on 30 August 2022)
Facetime (*n* = 1)	A cloud phone service. Key features include screen sharing, video or music sharing, and multi-user calls.	Interpersonal social media tools Mobile social media tools	https://support.apple.com/zh-cn/facetime (accessed on 30 August 2022)
Weblog Typepad (*n* = 1)	A class blog created by the teacher. Students can post their writings and exchange comments with teachers, peers, parents, etc. It is not clear which platform was used to create this class blog.	Text publishing tools	https://www.typepad.com/ (accessed on 1 September 2022)
School-built virtual learning environment called AMADIS (*n* = 1)	Each student has an account and a profile with their pictures on this platform. Students can post texts in a blog-like format and comment on each others’ posts. Students can also post pictures in addition to texts.	Text publishing tools	N/A (Internal platform, no public access)
Showme App (*n* = 1)	An application that allows users to record and create voice over presentations and share the presentations. Key features include recording voice, drawing, adding text, taking photos, and adding images. Sharing can be done online privately or to a community audience.	Mobile social media tools/ Audio Publishing tools	https://www.showme.com/about_showme (accessed on 27 August 2022)
Class Dojo (*n* = 1)	A digital platform that connects teachers, students, and families. Key features include: a classroom page where teachers can make announcements or post texts/photos about class activities to share with families and students, an e-portfolio section where students can upload their work to share, and a message function to communicate with families.	Text publishing tools Interpersonal social media tools/ Mobile social media tools	https://www.classdojo.com/zh-cn/?redirect=true (accessed on 27 August 2022)
Remind (*n* = 1)	An app for two-way communications among teachers, students, and parents. Key features include group messages, one-on-one messages, in-app translation, no revealing of personal contact information, and setting quiet hours/active hours.	Interpersonal social media tools Mobile social media tools	https://www.remind.com/ (accessed on 27 August 2022)
Papa (*n* = 1)	An audio publishing platform. Users can create personal profiles, record, upload, and share their audio or music of up to 6 min long accompanied by a picture that is either taken with the app, or chosen from the photo album. Users can like, leave a written or audio comment on others’ audios; they can also follow other users’ channels.	Audio publishing tools	http://papa.me/ (accessed on 29 August 2022)

**Table 4:** Application of social media tools in language and literacy development.

Author (Year)	First vs. Second Language	Dominant, Minority, or Foreign Language in the Country Where the Study Took Place	Social Media Tool Used	Purpose of Social Media Tool Integration	Target Aspect of Language and Literacy Development	Learning Activities Involving Social Media Tools
Dore et al. (2021) [52]	First language (73% of the sample) Second language (27% of the sample)	Dominant language	Zoom	To facilitate remote learning	Phonological awareness, letter knowledge, vocabulary, and narrative	The program was a virtual summer kindergarten readiness program which had five components: regular teacher–caregiver video chat; regular teacher–child video chat; a Watch Together activity where parents watched educational TV shows with their children; a Play Together activity where parents played with their children; and a Read Together activity where parents read books with their children. Zoom was used for conducting weekly meetings between teachers and caregivers and between teachers and children. ZIn the teacher–parent chat, teachers would review lessons from last week, provide an overview of the new materials and discuss the concepts covered in the new week. Caregivers could ask questions or discuss challenges they faced. In the teacher–child chat, teachers discussed the educational TV show that children had most recently watched with their parents in the Watch Together activity; then, the teacher read a book with the child and completed a learning activity (unspecified in the article). All activities in one chat session were supposed to support the same skill.
Ellison & Drew (2020) [53]	First language	Dominant language	Minecraft	To help students visualize the objects they will write about	Creative Writing	In this writing lesson, students were asked to write a setting description about a castle. Students used the Minecraft game to create their own castles in pairs. They were encouraged to discuss their building plans with their partners during the process. Then the castle designs were printed out from multiple angles and used as a stimulus for writing a setting description. However, the study did not mention whether any online interactions occurred.
Eubanks et al. (2018) [54]	Second language	Minority language	Book Creator	To provide an instructional tool that students can use to compose digital texts	Writing	In this 3-week 21st century writing workshop, each student were asked to create a story. They used the Book Creator app in the following steps in creating a digital story: first, they created three-part storyboards with narration, sketch, and digital media; second, they put all the materials together to create a digital book; third, they published the digital book to the digital classroom library and shared with parents.
Gaudreau et al. (2020) [62]	First language	Dominant language	Facetime	To provide tool for virtual communication, especially virtual Storytime practice	Story comprehension and vocabulary learning	The study took place in a lab environment. In a dialogic reading session, the experimenter read a book, gave prompts, and asked questions to a child over Facetime app on an iPad.
Genlott & Gronlund (2013) [55]	First language	Dominant language	Google Docs	To give students a real audience for their writing and let them know that they would receive feedback after their texts are published	Reading and writing	Google docs was used for publishing students’ written texts and receiving feedback from peers, teachers, and parents. Depending on the type of assignment and topic, students either wrote in a document that was shared with teachers and peers, or their work were published directly on a class web site which was built on Google Docs. It was mandatory that teachers and peers provided timely feedback after the texts were published.
Genlott & Gronlund (2016) [56]	First language	Dominant language	Google Drive (including Google Docs) and Google Sites	To provide a tool for collaborative learning, especially formative feedback and assessment	Reading and writing	The Write to Learn method contained seven steps in one learning cycle; GAFE tools were used to facilitate each step of the learning process: (1) Teacher published learning goals on Google Class Site. (2) Teacher published tutorials or inspirational videos on Google Class Sites to arouse students’ interests and provide them with pre-understanding of the task, which represented a flipped classroom approach. (3) Teacher provided written examples to illustrate the writing strategy and literacy genre of the task concerned in Google Drive for students to view and learn from. (4) Students started to write individually or in pairs and shared their writing via Google Drive with peers and teachers to give and receive formative feedback continuously. (5) Students learned to provide high quality formative feedback via practice using Google Drive; then they published the final version of what they wrote via Google Sites to get final feedback from more readers including other students, teachers, and parents. This helped increase students awareness and knowledge in writing for an audience. Publishing to a wider audience on Google Sites also ensured that students get final responses to the published texts. (6) Teacher evaluated student work to help the students see what learning goals they had accomplished and the next steps. All the past drafts, formative feedback available on Google Drive and Google Sites were taken into consideration when teachers evaluated student work.
Kaynar et al. (2020) [57]	Second language	Foreign language	Raz-Kids	To provide a platform for e-book reading	Reading, speaking, vocabulary	On Fridays, teachers would give assignments to students using the Raz-plus platform. Over the weekend, students read the assigned books, recorded their voices, completed associated learning activities, and sent them to teachers. On Monday, teachers would have a meeting with the students about the assignments and reward students for completion, such as by giving them extra stars that they could use to purchase icons for their avatars in the system. There was a comment function where teachers could either write or record a private message and sent to the students to comment on student work. After class, students could also use the platform as a self-assessment tool by reviewing books that were taught in class.
Lysenko & Abrami (2014) [58]	First language	Dominant language	ePearl	To develop self-regulated learning skills	Reading comprehension, listening comprehension, vocabulary, written expression	E-pearl was used in two ways. First, it was used in combination with another web-based application, ABRACADABRA (ABRA) which was an interactive multimedia tool for developing emerging literacy. ABRA housed 32 activities link to 26 interactive digital stories of various genres and 10 additional stories written by students and gamification features. When using ePearl together with ABRA, students can view the digital books on ABRA from within ePearl via a link to ABRA. Then they made recordings of excerpts of the books on ePearl, and created writings related to the stories on ePearl, posted illustrations from ABRA toghether with their writings on ePearl, reflected on their work and received feedback from teachers, parents, and peers. They could also set learning goals for all of these activities. Second, ePearl was also used independently. Students set learning goals, created digital artefacts including writings, drawings, pictures, and scanned and uploaded paper artefacts, reflected on their work, and received feedback from teachers, parents, and peers.
Miller (2018) [25]	Second language	Dominant language	Showme app Twitter	To enhance student engagement and establish an authentic audience for student work	Unspecified	(1) Showme App: students could take a picture of something that was related to their language learning, such as an object starting with the letter A when they learned the letter A. Then, the student put the picture in the ShowMe box and added a written and recorded description of the picture on the ShowMe app. Then, an email would be sent to family and classmates containing the picture, written description, and recording. (2) Twitter: teacher would regularly post links to students’ work and announcements on class twitter account for family and friends to see.
Szecsi & Szilagyi (2012) [63]	Heritage language	Minority language	Skype	To communicate with extended families and friends located in distant locations	Speaking, listening, reading, and writing	Skype was used for regular video chats with grandparents who were in Hungary. Communications included both verbal chats and typed chats. Grandma also read stories to Samuel over Skype once or twice a week and engaged in pretend play with Samuel. They pretended to be the characters in the stories and tell each other about their day.
Shin (2014) [38]	Second language	Dominant language	Weblog Typepad	To provide authentic and meaningful purposes for student writing by offering the students expanded audiences via blogging	Writing	The teacher initated a blog-mediated writing curriclum for English Language Arts (ELA) lessons. A class blog was created by the teacher on the Weblog Typepad. In their daily ELA lessons, students would first learn to write in different writing genres such as recounts, reports, and persuasive essays. Then students would post their writings on the class blog. The teacher invited other teachers, school librarians, her family members, and parents to join the blog as an expanded authentic audience for the students. The students and the audience could post and exchange comments on student writings on the class blog.
Silvia de Oliveira et al. (2013) [59]	First language	Dominant language	School-built virtual learning environment called AMADIS	To create a virtual literate environment for the students and promote social practices of writing and reading by allowing them to share with the entire school community	Reading and writing	(1) In a teacher-assigned project "Curiosity Awards", students could choose questions to investigate, such as “what did lions do besides attacking and eating?” Students would write journals (blog posts) on the virtual learning platform (VLE) to record their findings in both textual and picture formats, and to share their findings with peers and to give and receive comments. However, posting on the VLE is not a required component of the task. So, the amount that students posted on the VLE varied. (2) Students also posted and commented on the VLE spontaneously without involving a teacher-assigned task. They blogged and commented to ask questions, express themselves such as their interests, and to keep records.
Snell et al. (2022) [64]	First language (74% of the sample) Second language for the rest	Dominant language	Remind ClassDojo	To provide a home–school communication tool	Vocabulary	Teachers used Remind or ClassDojo to send weekly texts to parents that contained information on vocabulary words of the week and related activities that parents could try at home to help their children learn these vocabularies. These words were from books that were being read in the classroom in that week. Teachers could send the vocabulary and the activity texts together or separately. In the vocabulary text, there was also a link to a Text to Talk website. The website contained child-friendly definitions of the words, suggested activities, and a link to the book being read, if available. Note that this intervention was designed as a text intervention, so families without smartphones could receive the message as standard SMS messages. However, teachers all used social media tools to send the messages.
Theodosiadou & Konstantinidis (2015) [60]	First language	Dominant language	Google Drive (Google Slides)	To monitor pupil’s learning progress	Narrative, reflective and writing skills	A folder with a set of PowerPoint slides was created for each student on Google Drive. Each slide centered around one learning activity and had a picture related to that activity. Students wrote about their learning activities and posted their writing on the slides. Each slide had a consistent layout such that for each activity students were supposed to write about the following topics: (1) activity description; (2) new knowledge gained; (3) what they liked and why; (4) difficulties encountered and why. Consistent prompting questions were provided for each topic on each slide to facilitate students’ writing. Teachers and students discussed together in class to determine which activities the students should write about in the slides. When the e-portfolio was completed, students presented it to their parents.
Sun et al. (2017) [61]	Second language	Foreign language	Papa	To practice English speaking in a meaningful way	Speaking	Papa was used to complete oral assignments. Teachers would assign a specific question such as “what day is my best day?” for the children to record a response on Papa, and they were also asked to draw or find an image to accompany their recordings. Children recorded and uploaded their audio to Papa at home with the supervision of parents.
Olszewski & Cullen-Conway (2017) [65]	First language	Dominant language	Twitter	To deliver instructional materials to parents	Story comprehension, vocabulary, and print awareness	Twitter was used to deliver specially designed instructional videos on dialogic reading to parents. Videos less than 1 min were delivered to parents on Tuesday, Wednesday, and Thursday each week for 9 weeks. Videos demonstrated strategies across vocabulary, story comprehension, and print awareness. Each video started with a parent demonstrating the target strategy, followed by a brief description of the strategy by a narrator, and then ended with demonstration one more time.

**Table 5:** Effects of integrating social media tools on language and literacy development.

Author (Year)	Language and Literacy Skills Measured	Impact on Language and Literacy Development
Dore et al. (2021) [52]	Alphabet knowledge, emergent literacy skills including phonological awareness and print knowledge skills	Children exhibited small but significant improvements in alphabet knowledge, but no significant gains in phonological awareness or print knowledge skills.
Ellison & Drew (2020) [53]	A total writing quality score composed of three dimensions: text structure, sentence structure and grammatic features, vocabulary	Evidence was inconclusive regarding impact on creative writing ability. Among the six boys whose writing samples were scored, three boys’ pre-test and post-test scores were the same while the other three boys scored 1 or 2 points higher in the post-test.
Eubanks et al. (2018) [54]	Writing ability	The field investigator reviewed and evaluated the final published books and thought they were of A or A+ quality, but no details were given on the rating standards or procedures, or why the field investigator was qualified to make such judgements.In addition, field notes from observations showed that, compared to student writing in the same semester prior to the 21st century writing workshop, students (1) produced significantly more writing; (2) applied sentence structures learned before; (3) increased utilizations of new vocabulary in the writing workshop.
Gaudreau et al. (2020) [62]	Story comprehension including explicit and implicit comprehension and a page-by-page retell Vocabulary learning including expressive, receptive, and transfer vocabulary	Children in the video chat, face-to-face, and prerecorded book-reading groups performed similarly in story comprehension and vocabulary learning. However, children were more responsive to the dialogic prompts and questions in the video chat and face-to-face formats than in the prerecorded format.
Genlott & Gronlund (2013) [55]	Reading skillWriting skill	Reading Skill: the reading assessment tested the number of words that a child could read correctly in one minute. A slightly higher proportion of children in the experimental group (87.8%) achieved the pass level (at least 35 words per minute) than in the control group (84.7%). Moreover, 56% of the children in the experimental group achieved a high score (>55) while only 36% in the control group achieved a high score. Therefore, the authors concluded that the intervention resulted in more excellent readers in the experimental group. Writing skill: based on evaluations of students’ writings, the control group only produced very short texts which could hardly be considered as stories. On the contrary, the experimental group produced much longer stories which were clearer with a logical flow of events. Teachers read the students’ writing and claimed that test group students had mastered writing skills that were required by the national tests to be taken at grade 3, despite the fact that the participants were only in grade 1.
Genlott & Gronlund (2016) [56]	Reading and Writing	Students using the Writing to Learn (WTL) method scored significantly better than students who did not use the WTL method on the National Standard Tests (NST) of literacy. Moreover, there is no gender gap in literacy in the experimental group who used the WTL method.
Kaynar et al. (2020) [57]	N/A	Interviews with teachers showed that teachers believed that the platform enhanced students’ literacy skills, particularly fluency and accuracy in speaking and vocabulary growth.
Lysenko & Abrami (2014) [58]	Reading comprehension, listening comprehension, vocabulary, written expression	Students in the experimental group performed significantly better than students in the control group in terms of vocabulary and reading comprehension in both study 1 and study 2. The effect was consistent across grade levels, but no significant differences were found on listening comprehension. In study 2, the experimental group also scored significantly higher on written expression than the control group.
Miller (2018) [25]	N/A	Teachers believed that students’ talk became deeper and children were better able to express themselves as a result of using technology in the classroom.
Szecsi & Szilagyi (2012) [63]	N/A	Participants believed that their children made improvements in heritage language skills as a result of regular use of technologies. However, they also emphasized that adults’ creative and dedicated participation was required to achieve the optimal results.
Shin (2014) [38]	N/A	Linguistic analysis of the child’s blog postings showed the following achievements in literacy development: (1) The child became more aware of the interpersonal functions of text. He also displayed an understanding of the relationship between semiotic choices and meaning potentials which was above the writing proficiency of most second graders, as evidenced by his use of contradictory linguistic options in a recount about his experience going to amusement parks. (2) In his persuasive letter, the child demonstrated a level of understanding about the purpose and audience in constructing texts, which was beyond the proficiency and sophistication level required by the school curriculum standards for 2nd grade English Language Arts.
Silvia de Oliveira et al. (2013) [59]	Written language conceptualization	The findings were inconclusive. The number of children at the advanced stage of written language conceptualization, the alphabetic stage, increased from five to nine after using laptop with internet access for 5 months. Additionally, the number of children at the initial stage, the pre-syllabic stage, decreased from eight to six. No change was found for the intermediary 1 stage, the syllabic stage; and the number of children at the intermediary 2 stage, the syllabic alphabetic stage, actually decreased from three to one after 5 months.
Snell et al. (2022) [64]	Target word knowledge and receptive vocabulary	Children in the treatment group acquired significantly more target words than those in the control group, but there was no significant difference in the standardized measure of receptive vocabulary between the two groups.
Theodosiadou & Konstantinidis (2015) [60]	N/A	The author provided qualitative accounts on children’s improvements in literacy skills. Based on student work, they observed that students could only write very simple or unfinished sentences in the beginning; as they progressed through the project, they could write more carefully structured and detailed descriptions of each learning activity. Moreover, they would also express their dispositions or feelings for each activity and accompany their descriptions with meaningful rationale for their choices. Parental interviews also showed that parents thought their children became more articulate. Children became more willing to correct their spelling or syntax mistakes, as they knew their portfolio would also be presented to their parents.
Sun et al. (2017) [61]	Speaking skills: accuracy, fluency, and pronunciation	The experimental group made significantly larger gains in fluency, but gains in accuracy and pronunciation were comparable to that of the control group.
Olszewski & Cullen-Conway (2017) [65]	Story comprehension, vocabulary, print knowledge	Based on coding of observations, there were increases in parent dialogic reading strategies use during the treatment, when compared to the baseline. Children showed significant gains in vocabulary and story comprehension, but not on print knowledge from pretest to posttest.

## Data Availability

The data could be obtained upon request from the authors.
